# No sonographer, no radiologist: Assessing accuracy of artificial intelligence on breast ultrasound volume sweep imaging scans

**DOI:** 10.1371/journal.pdig.0000148

**Published:** 2022-11-23

**Authors:** Thomas J. Marini, Benjamin Castaneda, Kevin Parker, Timothy M. Baran, Stefano Romero, Radha Iyer, Yu T. Zhao, Zaegyoo Hah, Moon Ho Park, Galen Brennan, Jonah Kan, Steven Meng, Ann Dozier, Avice O’Connell

**Affiliations:** 1 Department of Imaging Sciences, University of Rochester Medical Center, Rochester, New York, United States of America; 2 Departamento de Ingeniería, Pontificia Universidad Católica del Perú, Lima, Peru; 3 Samsung Medison Co., Ltd., Seoul, Republic of Korea; 4 Samsung Electronics Co., Ltd., Seoul, Republic of Korea; 5 Department of Public Health, University of Rochester Medical Center, Rochester, New York, United States of America; Ludwig-Maximilians-Universität München Adolf-Butenandt-Institut: Ludwig-Maximilians-Universitat Munchen Biomedizinisches Centrum Munchen, GERMANY

## Abstract

Breast ultrasound provides a first-line evaluation for breast masses, but the majority of the world lacks access to any form of diagnostic imaging. In this pilot study, we assessed the combination of artificial intelligence (Samsung S-Detect for Breast) with volume sweep imaging (VSI) ultrasound scans to evaluate the possibility of inexpensive, fully automated breast ultrasound acquisition and preliminary interpretation without an experienced sonographer or radiologist. This study was conducted using examinations from a curated data set from a previously published clinical study of breast VSI. Examinations in this data set were obtained by medical students without prior ultrasound experience who performed VSI using a portable Butterfly iQ ultrasound probe. Standard of care ultrasound exams were performed concurrently by an experienced sonographer using a high-end ultrasound machine. Expert-selected VSI images and standard of care images were input into S-Detect which output mass features and classification as “possibly benign” and “possibly malignant.” Subsequent comparison of the S-Detect VSI report was made between 1) the standard of care ultrasound report by an expert radiologist, 2) the standard of care ultrasound S-Detect report, 3) the VSI report by an expert radiologist, and 4) the pathological diagnosis. There were 115 masses analyzed by S-Detect from the curated data set. There was substantial agreement of the S-Detect interpretation of VSI among cancers, cysts, fibroadenomas, and lipomas to the expert standard of care ultrasound report (Cohen’s κ = 0.73 (0.57–0.9 95% CI), p<0.0001), the standard of care ultrasound S-Detect interpretation (Cohen’s κ = 0.79 (0.65–0.94 95% CI), p<0.0001), the expert VSI ultrasound report (Cohen’s κ = 0.73 (0.57–0.9 95% CI), p<0.0001), and the pathological diagnosis (Cohen’s κ = 0.80 (0.64–0.95 95% CI), p<0.0001). All pathologically proven cancers (n = 20) were designated as “possibly malignant” by S-Detect with a sensitivity of 100% and specificity of 86%. Integration of artificial intelligence and VSI could allow both acquisition and interpretation of ultrasound images without a sonographer and radiologist. This approach holds potential for increasing access to ultrasound imaging and therefore improving outcomes related to breast cancer in low- and middle- income countries.

## Author summary

Breast cancer poses an urgent crisis in low- and middle-income countries, but the majority of the world lacks access to medical imaging for assessment of the breast. The use of volume sweep imaging (VSI) allows image acquisition of palpable breast lumps without an experienced sonographer. Images of breast lumps from VSI acquisitions can be interpreted by existing artificial intelligence to allow automatic and rapid diagnosis in areas without sonographers and radiologists. In this study, we fed expert-selected VSI images obtained by medical students with no prior ultrasound experience into an artificial intelligence (S-Detect) to test the potential for automatic diagnosis of palpable breast lumps. We found high diagnostic accuracy demonstrating a strong proof of concept for the potential of automatic diagnosis of palpable breast lumps without a radiologist or sonographer. Integration of artificial intelligence and VSI could increase access to diagnostic imaging around the world with the goal of improving global health.

## Introduction

Breast cancer is increasingly recognized as an urgent public health crisis in low- and middle-income countries (LMICs) secondary to a projected nearly 60% increase in both incidence and mortality of breast cancer over the last 20 years [1]. In contrast, in populations with access to screening mammography, there has been an observed decrease in breast cancer mortality [2–4]. It has been estimated that the majority of the nearly 2 million new cases of breast cancer every year will occur in LMICs [5]. The poor outcomes in LMICs owe, in part, to significant delays in breast cancer diagnosis (sometimes over 400 days) resulting in advanced disease at presentation [6]. Although diagnostic imaging is essential in the diagnosis of breast cancer, the majority of people in the world are thought to lack access to any diagnostic imaging [7,8]. Presumably, increased access to diagnostic imaging would be a first step in decreasing delays to diagnosis of breast cancer and improved outcomes.

Screening mammography is typically not an option in LIMCs for a variety of reasons [9,10]. Breast ultrasound, however, is a portable and low-cost imaging modality ideal for use in LMICs. In contradistinction to screening mammography which is performed on asymptomatic patients, ultrasound is typically used as a diagnostic technique to evaluate a patient presenting with a symptom, such as a palpable lump. Ultrasound and mammography are complementary imaging modalities each with their own strengths and weaknesses [11]. Since screening is often not available in LMICs, most patients with cancer will present with a palpable lump which is amenable to examination with ultrasonography [12]. In general, ultrasound is considered first-line evaluation for palpable breast abnormalities [13]. Despite its cost-effective and portable nature, ultrasound’s deployment has been limited by a lack of available ultrasound specialists for image acquisition and interpretation [8,14].

Volume sweep imaging (VSI) is an imaging technique that circumvents the problem of a lack of trained sonographers via the use of a standardized imaging protocol based on sweeps of the ultrasound probe over a target region in relation to external body landmarks [15]. VSI can be learned in a few hours by individuals without prior ultrasound training making ultrasound image acquisition readily accessible [16,17]. VSI has been combined with teleultrasound with encouraging clinical results for lung, obstetric, thyroid, and right upper quadrant scanning indications in Peru [15,18–21]. Clinical studies of breast and lung VSI have also shown promising results [22,23]. In the study of breast VSI, there was 97.6% agreement between VSI and standard of care ultrasound on mass presence and 87% agreement on BI-RADS assessments [22]. These results suggest that breast VSI could be deployed to rural areas to increase access to breast imaging. Nonetheless, a lack of interpreting providers and constraints on internet access bottleneck the impact of VSI as a radiologist is still needed to interpret the images.

Automatic interpretation of VSI with artificial intelligence would allow the performance of ultrasound imaging without any sonographer or radiologist. This has already been proposed and successfully tested for obstetric indications [24,25]. Such an automated system for breast scanning would offer a significant opportunity to increase access to breast imaging around the world. S-Detect (Samsung Medison, Seoul, Korea) is an artificial intelligence system developed as a tool for diagnostic analysis of breast masses [26]. It employs convolutional neural networks to arrive at a suggested diagnosis for a mass from a radiologist-detected lesion. S-Detect has shown excellent results in clinical testing (sensitivity of 85%, specificity of 95.4%, and diagnostic accuracy of 92.1%) highlighting its potential application in clinical practice [27]. Results consistently suggest S-Detect can perform at the same level or better than radiologists [27–32]. Therefore, this system would have significant potential to increase access to accurate diagnostic interpretations in the absence of a radiologist.

In this pilot study, we tested the performance of S-Detect on VSI ultrasound imaging performed by medical students without prior ultrasound experience with a portable ultrasound probe. The images tested were those obtained from the prior clinical study of breast VSI [22]. The goal of this study was to lay the theoretical groundwork for a fully automated system incorporating VSI and S-Detect. Comparison of the S-Detect VSI report was made to 1) the standard of care ultrasound report by an expert radiologist, 2) the standard of care ultrasound S-Detect report, 3) the VSI report by an expert radiologist, and 4) the pathological diagnosis. To our knowledge, S-Detect has never been previously tested on images obtained from a portable ultrasound device. Furthermore, previously, concerns had been raised regarding the use of S-Detect in cases where a sonographer is inexperienced or the ultrasound imaging was suboptimal, highlighting the importance of this investigation [26]. We hypothesized that S-Detect results between VSI and standard of care ultrasound would be similar given our experience with VSI and the literature surrounding S-Detect.

## Results

**Fig 1:**
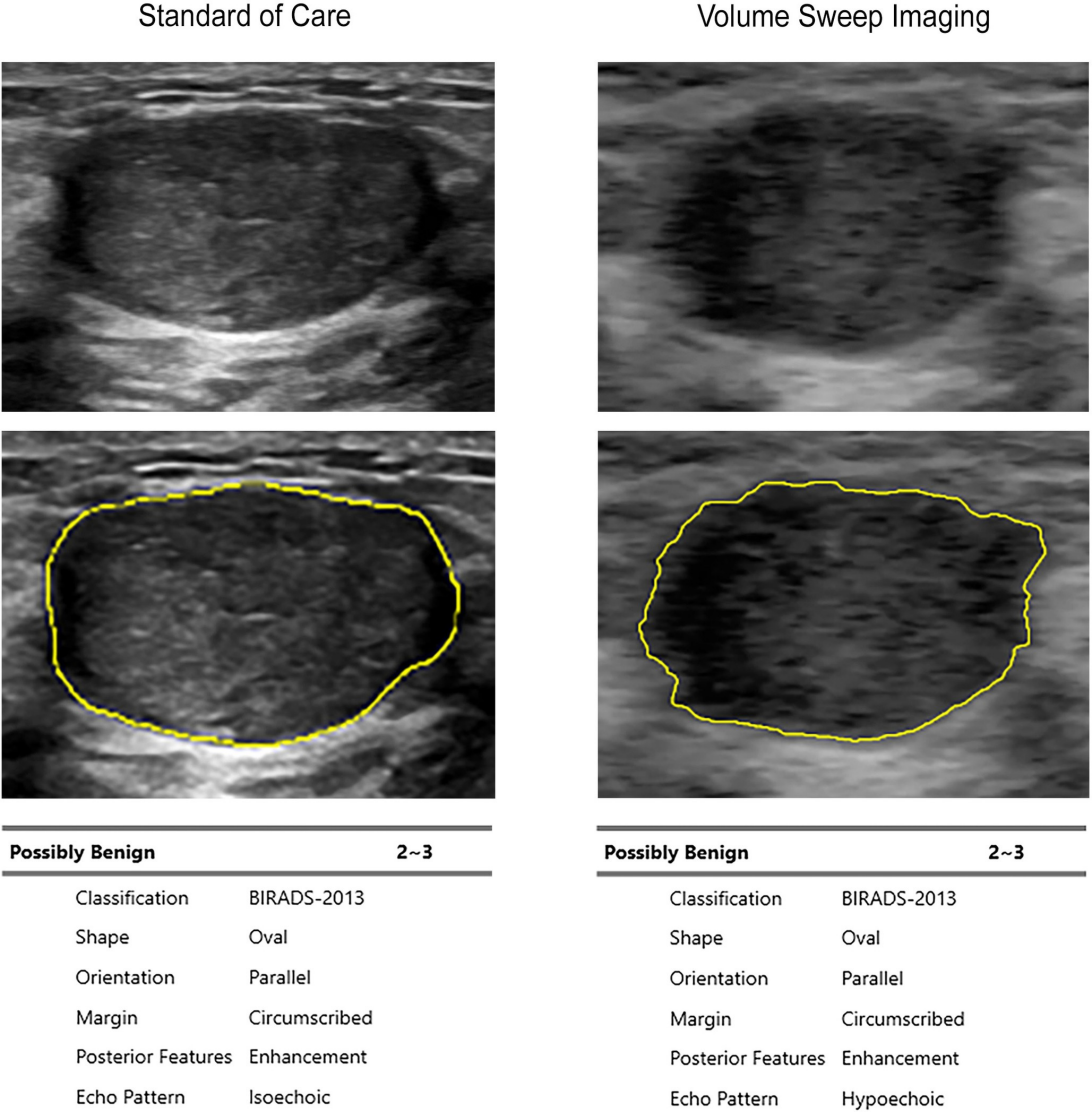
S-Detect Interpretation of a Fibroadenoma on Standard of Care and VSI. B-mode ultrasound images of a fibroadenoma are shown with their respective mass segmentations and S-Detect produced reports. The standard of care image was obtained by a certified expert breast sonographer using a General Electric Logiq E10 ultrasound machine. The VSI image was obtained by a medical student without prior medical experience after less than 2 hours of training on a portable Butterfly iQ ultrasound probe. S-Detect results between standard of care and VSI are identical apart from standard of care echogenicity reported as isoechoic (a trivial difference). The S-Detect results for VSI were identical to expert interpretation of standard of care and VSI.

**Fig 2:**
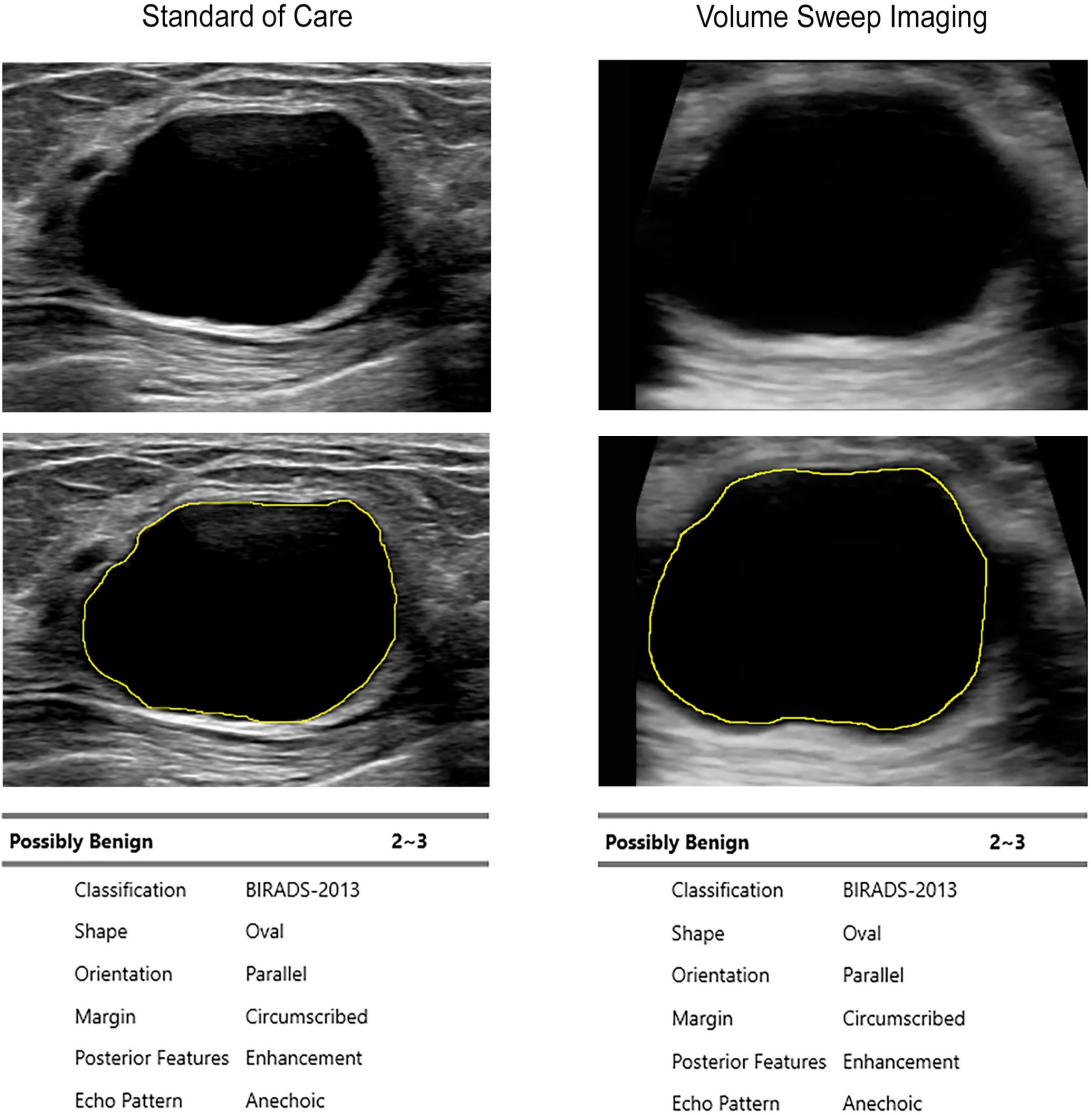
S-Detect Interpretation of a Cyst on Standard of Care and VSI. B-mode ultrasound images of a cyst are shown with their respective mass segmentations and S-Detect produced reports. The standard of care image was obtained by a certified expert breast sonographer using a Philips Epiq 7G ultrasound machine. The VSI image was obtained by a medical student without prior medical experience after less than 2 hours of training on a portable Butterfly iQ ultrasound probe. S-Detect results between standard of care and VSI are identical. The S-Detect results were also identical to expert interpretation of standard of care and VSI outside of mass shape which was called round on expert interpretation (a trivial difference).

**Fig 3:**
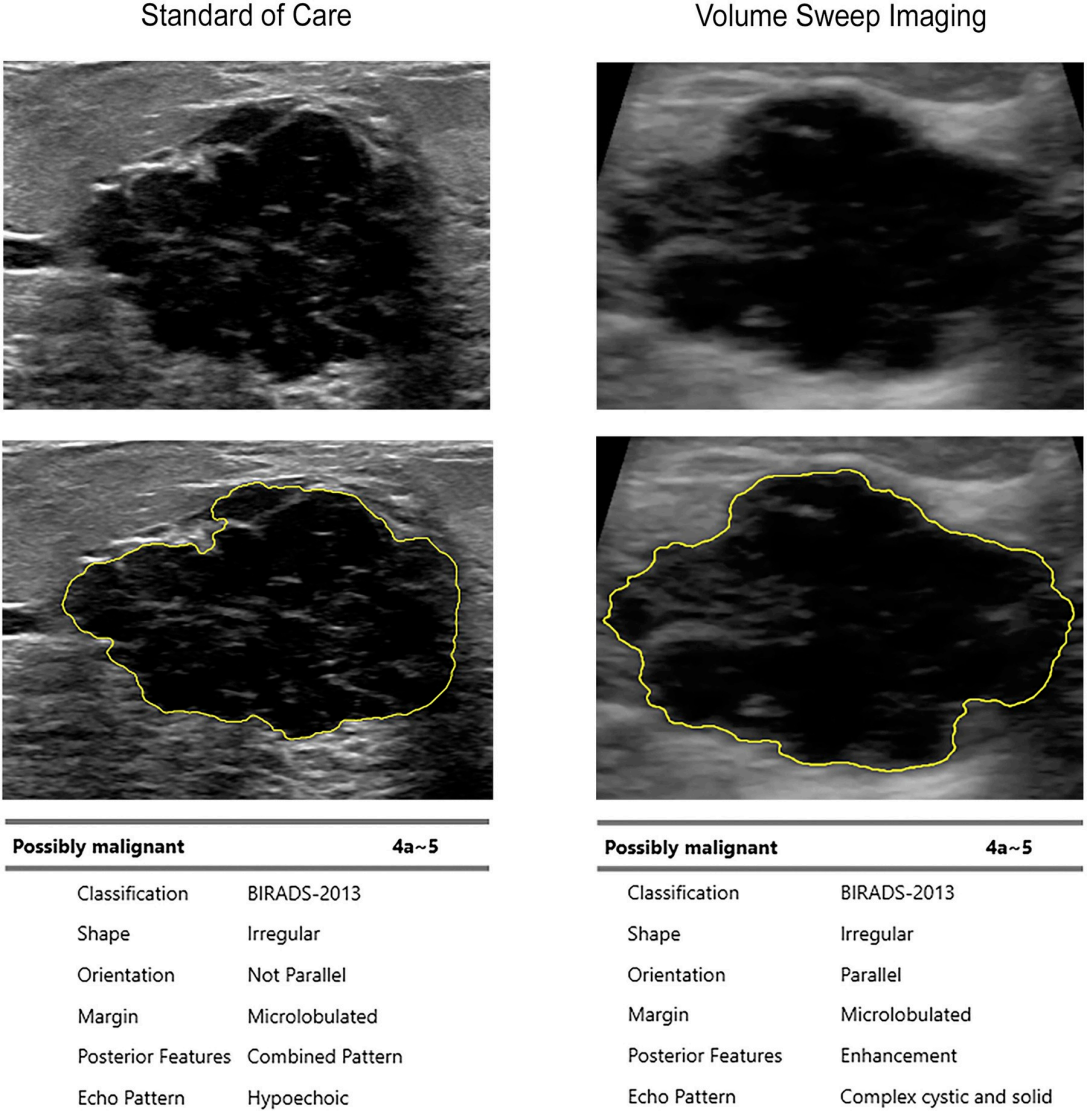
S-Detect Interpretation of Pathology Proven Invasive Ductal Carcinoma on Standard of Care and VSI. B-mode ultrasound images of invasive ductal carcinoma are shown with their respective mass segmentations and S-Detect produced reports. The standard of care image was obtained by a certified expert breast sonographer using a Philips Epiq 7G ultrasound machine. The VSI image was obtained by a medical student without prior medical experience after less than 2 hours of training on a portable Butterfly iQ ultrasound probe. S-Detect results between standard of care and VSI are similar with ultimate agreement on the “possibly malignant” interpretation. The S-Detect results were also similar to expert interpretation of standard of care and VSI with ultimate agreement on the “possibly malignant” characterization. The original BI-RADS assessment on both VSI and standard of care imaging expert interpretation was 4 (biopsy warranted). There was complete agreement across expert interpretation and S-Detect on microlobulated margins.

There were 115 masses analyzed by S-Detect from the curated data set (n = 13 abscesses, n = 20 cancers, n = 23 cysts, n = 6 fat necrosis, n = 24 fibroadenomas, n = 6 lipomas, n = 5 miscellaneous diagnoses, n = 8 sebaceous cysts, and n = 10 unknown) [22]. Patient demographics are seen in Table 1. Example S-Detect interpretations of a fibroadenoma, a cyst, and a cancer are seen in Figs 1–3. Among cancers, cysts, fibroadenomas, and lipomas, there was 87.7% agreement on diagnosis to both expert standard of care interpretation and expert VSI interpretation (Cohen’s κ = 0.73 (0.57–0.9 95% CI), p<0.0001). Within these same mass categories, there was 90.4% agreement on diagnosis between S-Detect interpretation of VSI and S-Detect interpretation of standard of care imaging (Cohen’s κ = 0.79 (0.65–0.94 95% CI), p<0.0001) and 90.5% agreement between the S-Detect VSI interpretation and the pathological diagnosis (Cohen’s κ = 0.8 (0.64–0.95 95% CI), p<0.0001). Table 2 shows full diagnostic agreement across different categories.

**Table 1:** Subject demographics and mass classifications. Categorical variables are summarized as n (proportion (95% CI)), while continuous variables are summarized as mean±standard deviation.

Metric	Category	Summary
Age (years)	-	42.9±16.4
Sex	Female	n = 110/115 (95.7% (90.1–98.6%))
	Male	n = 5/115 (4.35% (1.43–9.85%))
Race	African American	n = 25/115 (21.7% (14.6–30.4%))
	Asian American	n = 4/115 (3.48% (0.956–8.67%))
	Hispanic	n = 9/115 (7.83% (3.64–14.3%))
	White	n = 75/115 (65.2% (55.8–73.9%))
	American Indian or Alaskan Native	n = 1/115 (0.87% (0.022–4.75%))
	Other	n = 1/115 (0.87% (0.022–4.75%))
Laterality	Left breast	n = 64/115 (55.7% (46.1–64.9%))
	Right breast	n = 51/115 (44.3% (35.1–53.9%))
Quadrant	Upper outer	n = 56/115 (48.7% (39.3–58.2%))
	Lower outer	n = 13/115 (11.3% (6.16–18.6%))
	Upper inner	n = 21/115 (18.3% (11.7–26.5%))
	Lower inner	n = 14/115 (12.2% (6.82–19.6%))
	Retroareolar	n = 10/115 (8.7% (4.25–15.4%))
BMI (kg/m^2^)	-	29.8±8.24
How long has it been there? (days)	-	614±1350
Palpable?	Yes	n = 115/115 (100% (96.8–100%))
Pain?	Yes	n = 58/115 (50.4% (41–59.9%))
Discharge?	Yes	n = 14/115 (12.2% (6.82–19.6%))
Fever?	Yes	n = 4/115 (3.48% (0.956–8.67%))
Trauma?	Yes	n = 5/115 (4.35% (1.43–9.85%))
Classification	Abscess	n = 13/115 (11.3% (6.16–18.6%))
	Cancer	n = 20/115 (17.4% (11–25.6%))
	Cyst	n = 23/115 (20% (13.1–28.5%))
	Fat necrosis	n = 6/115 (5.22% (1.94–11%))
	Fibroadenoma	n = 24/115 (20.9% (13.9–29.4%))
	Lipoma	n = 6/115 (5.22% (1.94–11%))
	Miscellaneous	n = 5/115 (4.35% (1.43–9.85%))
	Sebaceous cyst	n = 8/115 (6.96% (3.05–13.2%))
	Unknown	n = 10/115 (8.7% (4.25–15.4%))
Pathology	Benign	n = 74/115 (64.3% (54.9–73.1%))
	Malignant	n = 20/115 (17.4% (11–25.6%))
	Indeterminate	n = 21/115 (18.3% (11.7–26.5%))
Pathology proven?	Yes	n = 38/115 (33% (24.6–42.4%))

**Table 2:** Agreement between S-Detect interpretation of malignancy on VSI, compared to other interpretations, sub-divided by the masses included in the analysis. SOC = standard of care.

Masses Included	Comparison Group	Overall Agreement (%)	Cohen’s kappa	P-Value
All	S-Detect SOC	79.1% (n = 91/115)	0.57 (0.42–0.72)	p<0.0001
	Expert SOC	73.9% (n = 85/115)	0.46 (0.29–0.62)	p<0.0001
	Expert VSI	75.7% (n = 87/115)	0.49 (0.33–0.66)	p<0.0001
	Pathological Diagnosis	75.5% (n = 71/94)	0.49 (0.3–0.67)	p = 0.00044
All minus fat necrosis, abscess, and sebaceous cyst	S-Detect SOC	87.5% (n = 77/88)	0.74 (0.59–0.88)	p<0.0001
	Expert SOC	84.1% (n = 74/88)	0.67 (0.51–0.83)	p<0.0001
	Expert VSI	85.2% (n = 75/88)	0.69 (0.54–0.85)	p<0.0001
	Pathological Diagnosis	84.3% (n = 59/70)	0.67 (0.49–0.85)	p<0.0001
Cancers, cysts, fibroadenomas, and lipomas	S-Detect SOC	90.4% (n = 66/73)	0.79 (0.65–0.94)	p<0.0001
	Expert SOC	87.7% (n = 64/73)	0.73 (0.57–0.9)	p<0.0001
	Expert VSI	87.7% (n = 64/73)	0.73 (0.57–0.9)	p<0.0001
	Pathological Diagnosis	90.5% (n = 57/63)	0.8 (0.64–0.95)	p<0.0001
Fibroadenomas	S-Detect SOC	87.5% (n = 21/24)	0.33 (-0.37–1)	p = 0.13
	Expert SOC	75% (n = 18/24)	0.27 (-0.23–0.78)	p = 0.31
	Expert VSI	75% (n = 18/24)	0.27 (-0.23–0.78)	p = 0.31
	Pathological Diagnosis	81.3% (n = 13/16)	0 (-1-1)	p>0.99
Cancers	S-Detect SOC	100% (n = 20/20)	*	-
	Expert SOC	100% (n = 20/20)	*	-
	Expert VSI	100% (n = 20/20)	*	-
	Pathological Diagnosis	100% (n = 20/20)	*	-
Cysts	S-Detect SOC	82.6% (n = 19/23)	0.4 (-0.13–0.93)	p = 0.086
	Expert SOC	87% (n = 20/23)	0 (-1.1–1.1)	p>0.99
	Expert VSI	87% (n = 20/23)	0 (-1.1–1.1)	p>0.99
	Pathological Diagnosis	86.4% (n = 19/22)	0 (-1.1–1.1)	p>0.99
Lipomas	S-Detect SOC	100% (n = 6/6)	†	-
	Expert SOC	100% (n = 6/6)	†	-
	Expert VSI	100% (n = 6/6)	†	-
	Pathological Diagnosis	100% (n = 5/5)	†	-

* Cohen’s kappa is undefined because all were rated malignant by all methods.

^†^ Cohen’s kappa is undefined because all were rated benign by all methods.

Table 3 shows the sensitivity and specificity for malignancy in relation to the pathological diagnosis. Among cancer, cysts, fibroadenomas, and lipomas, S-Detect interpretations of VSI had 100% sensitivity and 86% specificity for cancer. Table 4 shows agreement of S-Detect interpretation of mass characteristics of VSI in relation to S-Detect interpretation of standard of care imaging, expert VSI interpretation, and expert standard of care interpretation among cancers, cysts, fibroadenomas, and lipomas. In this analysis, there was greater than 80% agreement across S-Detect interpretations of VSI and S-Detect interpretations of standard of care imaging, expert VSI interpretation, and expert standard of care interpretation for mass orientation, the combined analysis of mass shape, the combined analysis of mass margins, and the combined analysis of mass echogenicity. S1 Table shows the full analysis of mass characteristics agreement across different categories of masses.

**Table 3:** Sensitivity and specificity for masses based on pathological diagnosis. SOC = standard of care.

Masses Included	Imaging Modality	Interpreter	Sensitivity	Specificity	PPV	NPV	Overall Agreement (%)	Cohen’s kappa	P-Value
All	VSI	Radiologist	100% (n = 20/20)	79.7% (n = 59/74)	57.1% (n = 20/35)	100% (n = 59/59)	84% (n = 79/94)	0.63 (0.45–0.8)	p<0.0001
	VSI	S-Detect	100% (n = 20/20)	68.9% (n = 51/74)	46.5% (n = 20/43)	100% (n = 51/51)	75.5% (n = 71/94)	0.49 (0.3–0.67)	p = 0.00044
	SOC	Radiologist	100% (n = 20/20)	75.7% (n = 56/74)	52.6% (n = 20/38)	100% (n = 56/56)	80.9% (n = 76/94)	0.57 (0.39–0.75)	p<0.0001
	SOC	S-Detect	100% (n = 20/20)	68.9% (n = 51/74)	46.5% (n = 20/43)	100% (n = 51/51)	75.5% (n = 71/94)	0.49 (0.3–0.67)	p = 0.00044
All minus fat necrosis, abscess, and sebaceous cyst	VSI	Radiologist	100% (n = 20/20)	80% (n = 40/50)	66.7% (n = 20/30)	100% (n = 40/40)	85.7% (n = 60/70)	0.7 (0.52–0.87)	p<0.0001
	VSI	S-Detect	100% (n = 20/20)	78% (n = 39/50)	64.5% (n = 20/31)	100% (n = 39/39)	84.3% (n = 59/70)	0.67 (0.49–0.85)	p<0.0001
	SOC	Radiologist	100% (n = 20/20)	76% (n = 38/50)	62.5% (n = 20/32)	100% (n = 38/38)	82.9% (n = 58/70)	0.64 (0.46–0.83)	p<0.0001
	SOC	S-Detect	100% (n = 20/20)	76% (n = 38/50)	62.5% (n = 20/32)	100% (n = 38/38)	82.9% (n = 58/70)	0.64 (0.46–0.83)	p<0.0001
Cancers, cysts, fibroadenomas, and lipomas	VSI	Radiologist	100% (n = 20/20)	88.4% (n = 38/43)	80% (n = 20/25)	100% (n = 38/38)	92.1% (n = 58/63)	0.83 (0.68–0.97)	p<0.0001
	VSI	S-Detect	100% (n = 20/20)	86% (n = 37/43)	76.9% (n = 20/26)	100% (n = 37/37)	90.5% (n = 57/63)	0.8 (0.64–0.95)	p<0.0001
	SOC	Radiologist	100% (n = 20/20)	83.7% (n = 36/43)	74.1% (n = 20/27)	100% (n = 36/36)	88.9% (n = 56/63)	0.77 (0.6–0.93)	p<0.0001
	SOC	S-Detect	100% (n = 20/20)	83.7% (n = 36/43)	74.1% (n = 20/27)	100% (n = 36/36)	88.9% (n = 56/63)	0.77 (0.6–0.93)	p<0.0001

**Table 4:** Agreement on mass characteristics between S-Detect interpretation of VSI, compared to other interpretations, sub-divided by the masses included in the analysis. The combined mass shape analysis is between “round/oval” or “irregular.” The combined margin analysis is between “circumscribed” or “non-circumscribed.” The combined echogenicity analysis is between “anechoic/hypoechoic/complex cystic or solid,” “isoechoic/hyperechoic,” or “heterogenous.” SOC = standard of care.

Masses Included	Metric	Comparison Group	Overall Agreement (%)	Cohens kappa	P-Value
Cancers, cysts, fibroadenomas, and lipomas	Mass Orientation	S-Detect SOC	80.8% (n = 59/73)	0.45 (0.2–0.71)	p = 0.0013
		Expert SOC	87.7% (n = 64/73)	0.57 (0.3–0.83)	p<0.0001
		Expert VSI	84.9% (n = 62/73)	0.52 (0.26–0.78)	p<0.0001
	Mass Shape	S-Detect SOC	76.7% (n = 56/73)	0.54 (0.34–0.73)	p<0.0001
		Expert SOC	60.3% (n = 44/73)	0.34 (0.15–0.52)	p = 0.0015
		Expert VSI	60.3% (n = 44/73)	0.32 (0.12–0.51)	p = 0.0014
	Mass Shape (combined)	S-Detect SOC	86.3% (n = 63/73)	0.65 (0.44–0.85)	p<0.0001
		Expert SOC	78.1% (n = 57/73)	0.48 (0.25–0.7)	p = 0.00039
		Expert VSI	79.2% (n = 57/72)	0.48 (0.24–0.71)	p = 0.00018
	Mass Margins	S-Detect SOC	63% (n = 46/73)	0.31 (0.1–0.5)	p<0.0001
		Expert SOC	64.4% (n = 47/73)	0.29 (0.075–0.51)	p = 0.00031
		Expert VSI	67.1% (n = 49/73)	0.35 (0.13–0.56)	p<0.0001
	Mass Margins (combined)	S-Detect SOC	82.2% (n = 60/73)	0.61 (0.42–0.8)	p<0.0001
		Expert SOC	80.8% (n = 59/73)	0.56 (0.35–0.77)	p<0.0001
		Expert VSI	84.9% (n = 62/73)	0.65 (0.46–0.84)	p<0.0001
	Mass Echogenicity	S-Detect SOC	56.9% (n = 41/72)	0.38 (0.21–0.54)	p<0.0001
		Expert SOC	57.5% (n = 42/73)	0.36 (0.19–0.53)	p<0.0001
		Expert VSI	57.5% (n = 42/73)	0.37 (0.2–0.54)	p<0.0001
	Mass Echogenicity (combined)	S-Detect SOC	93.1% (n = 67/72)	0.64 (0.34–0.94)	p<0.0001
		Expert SOC	100% (n = 73/73)	1 (1–1)	p<0.0001
		Expert VSI	98.6% (n = 72/73)	0.9 (0.71–1.1)	p<0.0001
	Mass Posterior Acoustic Features	S-Detect SOC	64.4% (n = 47/73)	0.47 (0.31–0.64)	p<0.0001
		Expert SOC	75.3% (n = 55/73)	0.59 (0.43–0.76)	p<0.0001
		Expert VSI	76.7% (n = 56/73)	0.62 (0.47–0.78)	p<0.0001

Among pathologically proven cancers (n = 20), S-Detect rated all as “probably malignant” on both standard of care imaging and VSI. Among pathologically proven fibroadenomas (n = 8), there was 88% agreement with S-Detect interpretation of standard of care imaging (n = 7/8) and 63% agreement with S-Detect interpretation of VSI (n = 5/8). Among the entire category of masses classified as fibroadenoma either by pathology or imaging stability there was 92% agreement with S-Detect interpretation of standard of care imaging (n = 22/24) and 88% agreement with S-Detect interpretation of VSI (n = 21/24). Among cysts, there was 78% agreement with S-Detect interpretation of standard of care imaging (n = 18/23) and 87% agreement with VSI S-Detect interpretation (n = 20/23).

## Discussion

**Fig 4:**
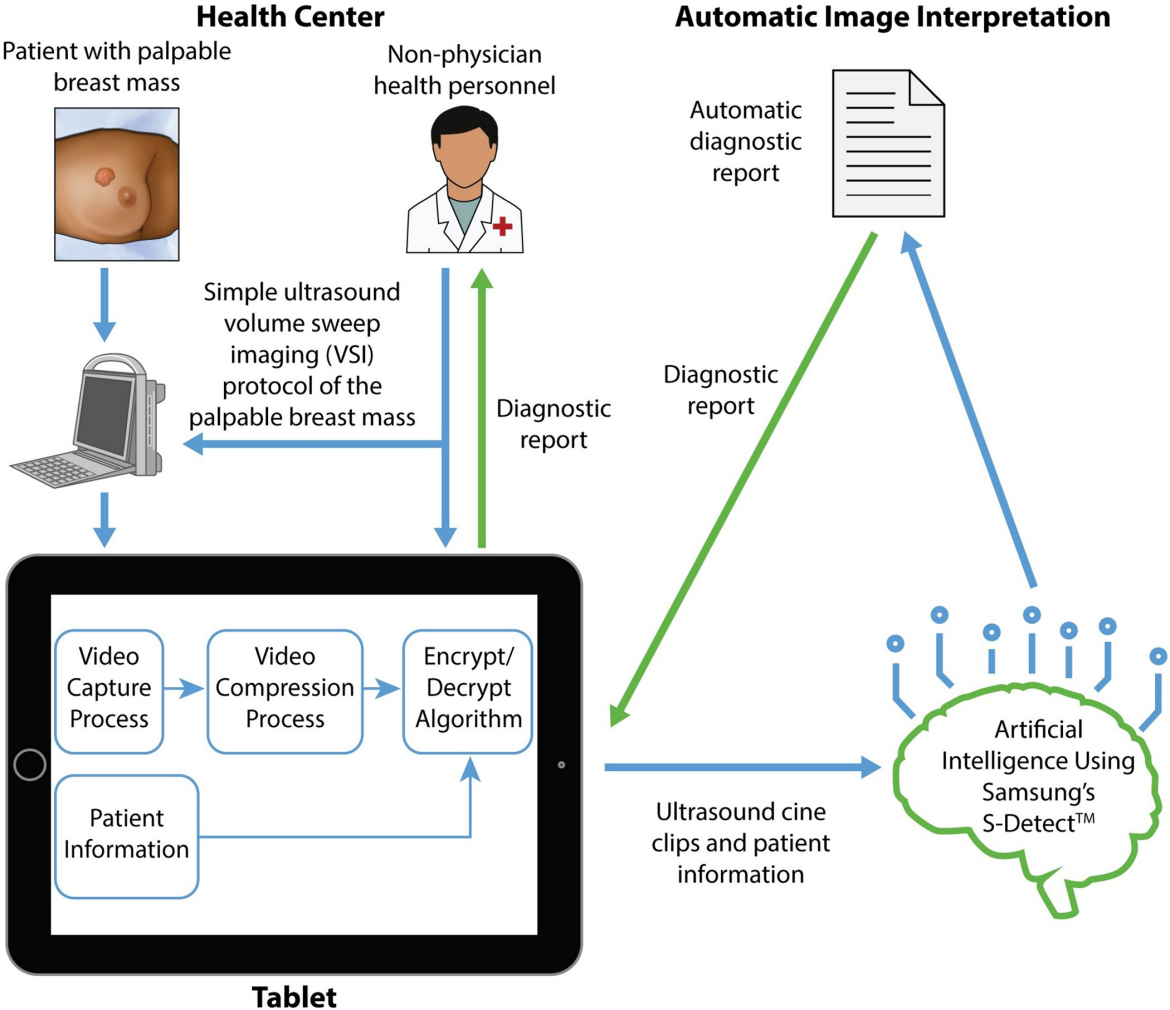
Proposed Model for Integration of Breast VSI and Artificial Intelligence. Schematic demonstrating the integration of VSI and artificial intelligence. In this model, a patient presenting with a palpable breast mass would be scanned by an individual with a few hours of ultrasound training using the VSI protocol. Imaging acquisitions would be funneled into artificial intelligence software that outputs a diagnostic report. The diagnostic report would then be available for the health center and patient. This would result in rapid and automated diagnosis without a radiologist or a sonographer.

The results from our study showed high diagnostic accuracy of the S-Detect interpretation of VSI in relation to expert radiologist interpretation of standard of care imaging, S-Detect interpretation of standard of care imaging, expert radiologist interpretation of VSI, and the pathological diagnosis. The diagnostic accuracy of S-Detect was overall similar to prior published studies, performing on par with mammography [27,33]. The high agreement observed suggests the portable ultrasound image quality was not an obstacle to S-Detect interpretation. Therefore, with further work packaging the systems together, we believe VSI could be incorporated with S-Detect to produce an ultrasound system that would require neither an experienced sonographer nor interpreting radiologist to automatically and rapidly diagnose palpable breast masses (Fig 4). Such a system would simply require a few hours of training on the VSI protocol, a tablet equipped with the diagnostic software, and a portable ultrasound probe. Given the urgency and challenges posed by breast cancer in LMICs, such a system could assist in decreasing delays to diagnosis with the potential of improving outcomes. In many settings, the sobering alternative to automatic diagnosis may be no imaging at all highlighting the potential value of this approach.

S-Detect showed excellent diagnostic performance across cysts, fibroadenomas, lipomas, and cancer. In all cases of malignancy, a rating of “possibly malignant” was produced which would result in a patient being referred for higher care and biopsy. There were three cases in which S-Detect referred to a cyst on VSI as “possibly malignant” which could be addressed in future updates of the program. Analysis of the VSI cyst results showed that the VSI images of those cysts likely did not have enough margin around the region of interest to allow accurate detection as S-Detect needs surrounding breast parenchyma to make an accurate diagnosis. After adding randomized noise around the cyst, benign results were able to be obtained in all cases. Similarly, a few cases of cysts on standard of care imaging were also reported as malignant. The standard of care cyst results were thought to relate to differences in the machines used to train S-Detect and would require further training and modification of S-Detect to produce benign results.

Apart from these few cases, expert analysis suggested that all results of S-Detect on the VSI exams were otherwise justifiable. In some cases, masses straddle the BI-RADS 3 or 4 categories making biopsy or follow-up reasonable. Similarly, though a mass may return pathologically benign, in many cases the suspicious features still warranted biopsy. The disagreements observed in mass characteristics likely related to the known accepted large inter-reader differences in interpretation of breast imaging that have been previously documented [32,34,35]. Many of the disagreements on mass characteristics were thought to be trivial in nature (i.e. calling a mass round or oval). Other disagreements were thought to relate to the S-Detect algorithm’s performance on images from different ultrasound machines and/or general error in expert interpretation.

These results suggest that this artificial intelligence is indeed ready to deploy in the near future. Even conceding a few benign lesions being considered “possibly malignant,” the authors believe the 100% detection of malignancy in this sample would easily negate any additional healthcare costs and anxiety associated with the few scattered false positive results which would be promptly reconciled on referral and/or biopsy. The false negative is the truly worst outcome possible in this dynamic as it would result in delay to diagnosis and potentially significant increased morbidity. In our sample, there were no cases of false negatives. Since S-Detect is already very high performing, future updates to the program algorithm should only increase its accuracy further.

The primary clinical advantage of this approach is facilitating increased access to ultrasound imaging which could decrease the often substantial delays to diagnosis of breast cancer associated with advanced disease along with increased morbidity and mortality [6,36]. While more than 70% of breast cancer cases present in stage 1 or 2 in high-income settings, it is estimated that only 20–50% of breast cancer cases present in stage 1 or 2 in LMICs highlighting the need to detect breast cancer earlier in these settings [37]. In cases of benign breast lesions, the automatic diagnostic framework would allow reassurance and potentially save the patient costs associated with workup including any associated travel expenses to a site with breast imaging capacity.

Despite the high incidence and mortality of breast cancer in LMICs, only 5% of global spending on cancer is currently allocated to those settings [38]. Automatic and rapid VSI breast ultrasound acquisition and diagnosis could be one way to help bridge the gap until advanced imaging services are more readily available. Advanced imaging services such as mammography are faced with substantial challenges to implementation in lower-resource settings [9,10]. Therefore, cost-effective and sustainable solutions to increase imaging access in LMICs should be considered [1,39–41]. Our proposed automated diagnostic framework (Fig 4) holds much potential in this regard, but from a public health perspective, studies will need to be conducted how best to market this technology to the community and integrate it into existing healthcare systems. Factors such as availability of biopsy services and surgical/oncological services may somewhat limit the initial impact of deployment at this present time.

There is further work to be done in order to create and integrate an automatic diagnostic framework for breast masses into clinical practice. S-Detect requires that a DICOM image be input into the program. In this study, all of the VSI frames selected for interpretation by S-Detect were chosen by an expert breast imager. This is a workflow and expertise restriction which may be eliminated in the near future by additional artificial intelligence systems trained to automatically detect the mass within the VSI frame and input it into S-Detect. Also, in general, artificial intelligence such as S-Detect could benefit from considering clinical parameters such as age and family history of breast cancer [31]. We noted in this study that abscesses and sebaceous cysts were suboptimally analyzed with S-Detect which is not surprising given that the clinical history is crucial for an experienced breast imager to make an accurate diagnosis. While it is true these cases are usually without clinical ambiguity negating the need for automated diagnosis, input of clinical history could favor a benign diagnosis if infectious symptom are present. Similarly, the artificial intelligence could be upgraded to expand from simply calling masses “possibly benign” or “possibly malignant.” It would be possible to have Breast Imaging-Reporting and Data System (BI-RADS) assessments or a suspected diagnosis (i.e. fibroadenoma or cyst) included in the final assessment.

Breast VSI in this form is currently limited to scanning of palpable abnormalities, not general screening. Automated approaches to whole breast ultrasound screening have been previously described but are not generally cost-effective or immediately deployable in rural areas [42]. As a theoretical possibility, S-Detect could also be integrated with whole breast screening ultrasound systems to produce automated diagnosis without any human operator, but again, costly systems would limit this approach’s relevance to LMICs. VSI could possibly be adjusted to completely scan the entire breast in future iterations, but this approach would be limited by many issues such as potentially suboptimal image quality, false positives, false negatives, and increased time to perform the exams. Palpable abnormalities are ideal for VSI examinations as they are often superficial and large in size allowing ease of diagnosis. Furthermore, the most common presenting feature of breast cancer is a palpable mass, and virtually all breast cancer in LMICs will present as a palpable lump [12,43]. Given the current reported delays to diagnosis for palpable lumps, a system for exclusive automatic diagnosis of palpable lumps is of significant value even though it will inherently not detect asymptomatic lesions which require mammographic examination or other advanced imaging.

One limitation of the study is that our analysis is limited to one single frame selected from the VSI cine clips for interpretation. Future expansions could be made to test multiple frames from a VSI clip to better characterize the performance of S-Detect, especially when a mass is only partially visualized on a VSI frame. Nonetheless, the goal of this study was to demonstrate a proof-of-concept minimizing the impact of this limitation. We approached the study with the interrelated goals of assessing S-Detect performance on both a portable ultrasound machine and on VSI scans. It is possible that S-Detect would perform better given an experienced operator acquiring the images on the portable ultrasound machine. Our study design precluded assessment of this possibility. However, as the ultimate goal of this study was to lay the groundwork for a system that could be used in rural areas, this limitation is of minimal significance to the conclusion that S-Detect functions well with scans obtained on a portable machine. In addition, the already high agreement with standard of care suggests that any increased performance of S-Detect on portable scans obtained by an expert sonographer could only be marginal at best.

## Conclusion

The global community is facing an urgent public health crisis with increasing incidence and mortality of breast cancer in LMICs. The lack of readily available ultrasound imaging likely contributes to significant delays to diagnosis of palpable masses, presumably resulting in increased morbidity and mortality. New approaches are needed to increase access to medical imaging in cost-effective and sustainable ways. The integration of volume sweep imaging and artificial intelligence is a promising avenue to realize increased access to imaging. Our synthesis of an inexpensive, portable ultrasound scanner with VSI and artificial intelligence would allow for automatic rapid image acquisition and preliminary but actionable interpretation without a dedicated sonographer or radiologist. In this study, we demonstrated excellent diagnostic accuracy of S-Detect on VSI scans performed by medical students without prior ultrasound training after less than 2 hours of instruction. Further work is needed to synthesize the individual components of this system and automate the identification of a breast lump in a VSI clip for analysis by artificial intelligence. In the context of this urgent public health crisis, we propose expedited further study of this potentially life-saving innovation. Compared to current practice, this technology holds the potential to reduce cost by at least an order of magnitude while increasing accessibility and providing a preliminary, actionable diagnosis to millions of underserved patients.

## Methods

### Ethics statement

This study was approved by the University of Rochester Research Subjects and Review Board (Study 00005262). Formal written consent was obtained from every participant.

### Breast volume sweep imaging

**Fig 5:**
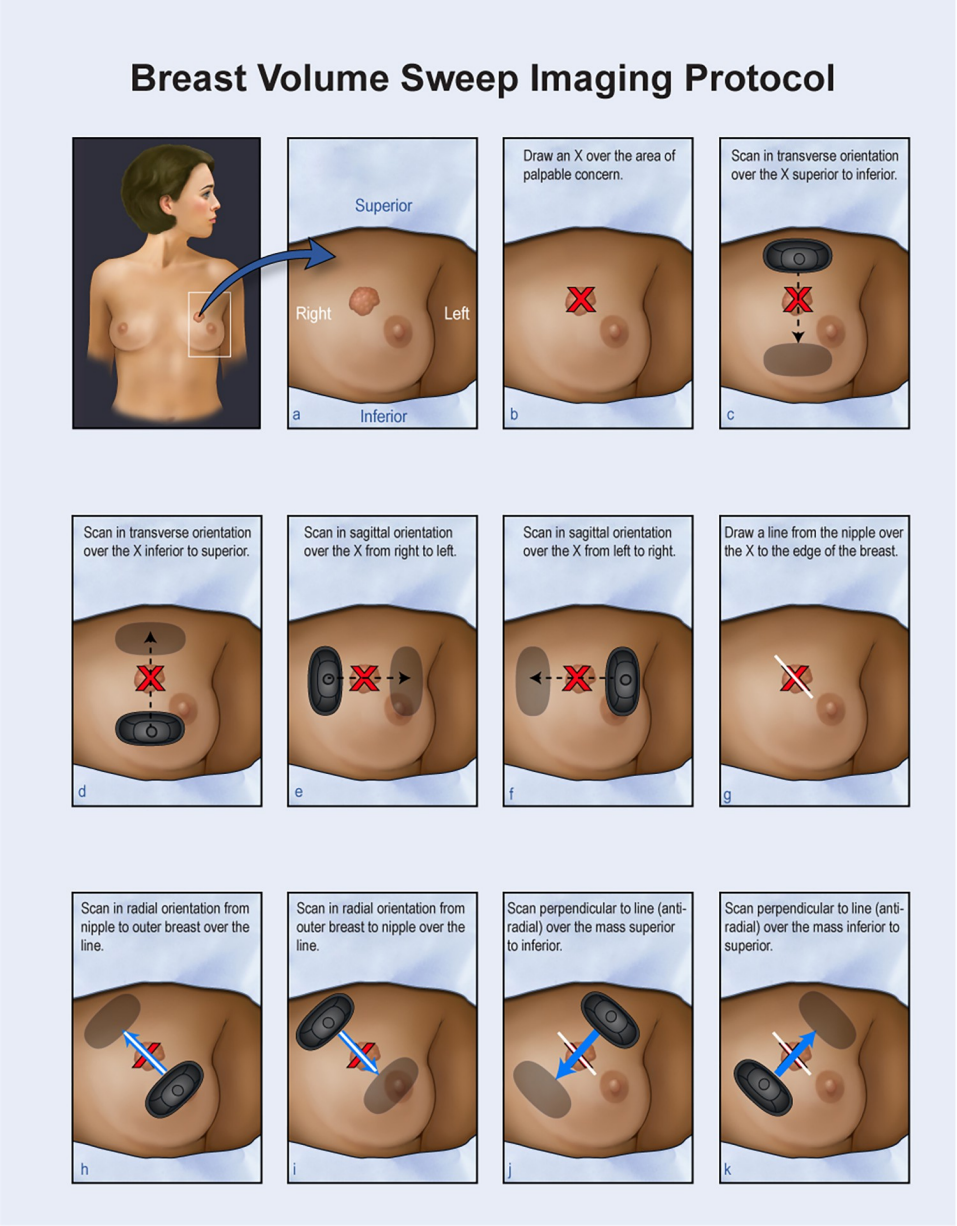
Breast VSI Poster. Illustration of how to perform breast VSI. Sweeps of the ultrasound probe are performed in the transverse, sagittal, radial, and anti-radial orientations.

VSI was designed to increase access to imaging in areas with a lack of medical professionals [15]. In VSI, the ultrasound probe is swept over a target region in relation to easily recognized external body landmarks using presets of the ultrasound probe removing the need for anatomical knowledge or technical ultrasound ability. The sweeps are saved as video clips which are later interpreted by a specialist. VSI can be learned over the course of hours as opposed to the years of dedicated training often required for ultrasound specialists [16,17]. This approach has already been successfully piloted for lung, obstetric, right upper quadrant, and thyroid scanning [15,18–21,23]. Specifically, breast VSI has been successfully clinically tested with encouraging results [22]. At this time, breast VSI is only indicated for evaluation of palpable abnormalities. It is generally accepted that ultrasound is first-line evaluation for palpable breast abnormalities [13,44–46]. In breast VSI, the operator marks a palpable abnormality with an “X” and sweeps the probe over the abnormality in several orientations (Fig 5). The protocol can be performed within 5 minutes. It should be emphasized the individual acquiring the images is not adjusting the ultrasound machine or interpreting the images in any way. VSI operators are instead encouraged to focus on their probe technique maintaining good contact with the skin and holding the probe steady.

### S-Detect

**Fig 6:**
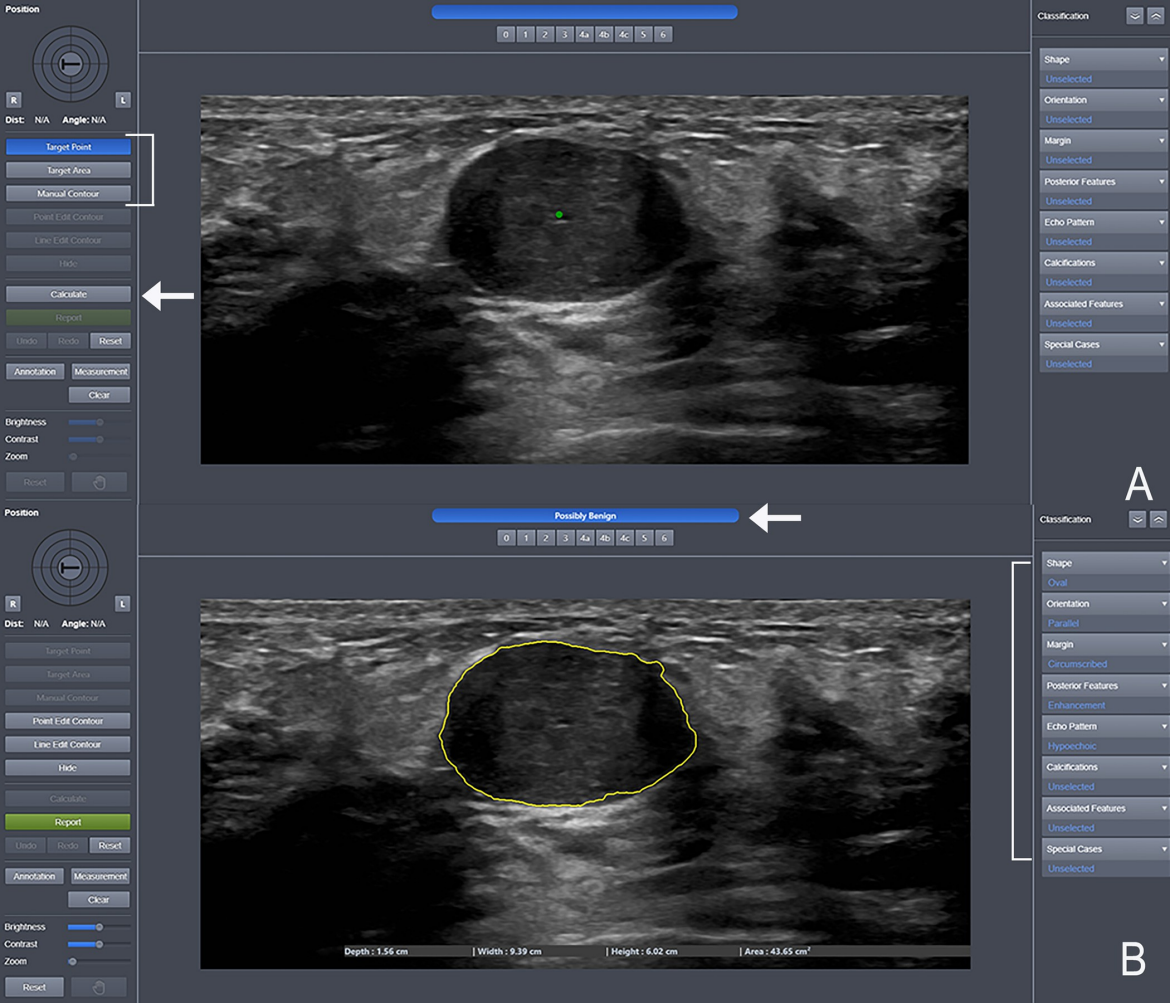
Screenshots of S-Detect User Interface. (A) Screenshot showing the S-Detect user interface. There are three possible methods to target the mass in S-Detect (bracket). In this screenshot, the “Target Point” method has been used. This method involves placing a single point within the target mass (green dot). This could also be accomplished using a “Target Area” or manually outlining the mass boundaries using the “Manual Contour” option. Once the mass has been targeted, S-Detect will produce an interpretation after pressing the “Calculate” button (arrow). (B) Screenshot showing the results after a mass has been targeted. S-Detect outputs a result of “possibly benign” or “possibly malignant” (arrow) as well as a list of mass characteristics (bracket). The mass segmentation is outlined in yellow and this can be adjusted or redone if necessary.

S-Detect is a computer-aided diagnosis system originally designed as an assistance tool for radiologists. A PC-based research version of S-Detect was used for this study. DICOM images are input into S-Detect which outputs mass characteristics and a dichotomous assessment of “possibly benign” or “possibly malignant” (Fig 6). S-Detect always outputs a result for all possible categories regardless of the image quality. In the current version of S-Detect, a mass is required to be identified by the user. Once the mass has been identified, the contours of the mass can be subsequently edited if needed. The current version of S-Detect does not distinguish between images with a mass or without a mass. Therefore, future integration with VSI will require additional software to identify a mass within the breast tissue.

### Study design

The masses in this study were obtained from a curated data set from a prior clinical study of breast VSI [22]. All breast VSI examinations were performed by medical students without prior ultrasound experience after less than two hours of training. The VSI scans were performed on the hand-held Butterfly iQ ultrasound probe at the University of Rochester (Butterfly Network, Guilford) with its small organ preset. VSI operators were blinded to the clinical history and were explicitly instructed not to look at the ultrasound images while scanning as an additional safeguard. Standard of care ultrasound examinations were performed in accordance with conventional clinical practice by experienced certified breast sonographers. Standard of care scans were performed on either a Logiq e10 (General Electric, Boston) or an Epiq 7G (Philips, Amsterdam) ultrasound machine with a high-frequency probe with settings adjusted across exams to obtain optimal imaging. The iQ costs approximately $2,000 while the state-of-the-art standard of care ultrasound machines are approximately 50–75 times more expensive.

Individuals over 18 years of age of either sex were eligible to enroll provided they felt a palpable breast lump. For the purposes of this investigation into the accuracy of S-Detect on VSI scans, subjects were eligible for analysis only if VSI and standard of care imaging both showed a sonographic mass as S-Detect does not provide interpretations of normal breast tissue. A VSI frame best representing the mass per expert analysis was chosen by a radiologist and converted to DICOM image format. This DICOM file along with a DICOM file from the standard of care exam were input into S-Detect. The S-Detect interpretation output included both mass characteristics and a final assessment. Mass characteristics included mass orientation (“parallel” or “not parallel”), mass shape (“round,” “oval,” or “irregular”), mass margins (“circumscribed,” “indistinct,” “microlobulated,” “angular,” or “spiculated”), mass echogenicity (“anechoic,” “hypoechoic,” “isoechoic,” “hyperechoic,” “complex cystic and solid,” or “heterogeneous”), and mass posterior acoustic features (“none,” “enhancement,” “shadowing,” or “combined”). The final assessment was “possibly benign” or “possibly malignant.”

The expert VSI exam interpretation procedure was previously described in the clinical study of breast VSI [22]. VSI expert interpretations were made from the best frames/sweeps available by a specialist in breast imaging with over 20 years of experience. The standard of care exam reports were produced per normal clinical practices by an unblinded breast imager. There was a similar reporting scheme employed for the expert interpretations to the one used by the artificial intelligence with two notable differences. The first is that S-Detect will always output a result even if the feature is suboptimally visualized. In the VSI scan expert readings, if one of these features was not visualized or non-diagnostically assessed, it was reported as non-visualized. In standard of care examinations, all features were always visualized. The second difference was the expert VSI and standard of care reports used discrete BI-RADS assessments as opposed to the “possibly benign” or “possibly malignant” categorization used by S-Detect.

BI-RADS is an important classification tool in breast imaging which provides a standard format for interpretation and reporting considered standard of care [47,48]. BI-RADS assessments for standard of care examinations apply to the entirety of the breast tissue examined whereas VSI is solely examining a single palpable location. Therefore, standard of care BI-RADS assessments were adjusted to account for only the palpable finding disregarding findings related to any other breast tissue routinely imaged as part of the standard of care exam. These adjusted assessments also disregarded other clinical history related to prior imaging. BI-RADS assessments were subsequently converted to “probably benign” (BI-RADS 2 or 3) or “probably malignant” (BI-RADS 4 or 5) for analysis. In clinical practice, BI-RADS 2 represents a finding with 100% chance of benignity such as a simple cyst and requires no follow-up. BI-RADS 3 represents a finding with less than a 2% chance of malignancy and is typically managed with a 6-month follow-up. Both BI-RADS 4 (2–95% chance of malignancy) and 5 (>95% chance of malignancy) are typically managed with biopsy.

Among the 170 lumps scanned in the prior clinical trial of breast VSI, n = 115 sonographic masses were identified on VSI and n = 119 sonographic masses were identified on standard of care. This discrepancy was due to 4 sonographic masses seen on standard of care imaging and not on VSI. This was further explored in the prior publication but related to subtle lesions likely of no clinical significance [22]. Since this is a pilot trial, we therefore only tested sonographic masses identified on VSI. The other lumps in the prior study corresponded to palpable areas without a corresponding sonographic mass which is a common occurrence. Therefore, these could not be tested in S-Detect since there was no sonographic mass to test. As explained earlier, this version of S-Detect requires the user to identify a mass which is subsequently fed to S-Detect for analysis. S-Detect does not distinguish background breast parenchymal tissue from sonographic breast masses.

We conducted several analyses of the of agreement including across all masses. However, abscess, sebaceous cysts, and fat necrosis often depend on the clinical history to be distinguished from malignancy. Therefore, we conducted a separate analysis removing these diagnoses from consideration. Additional analysis was performed solely for fibroadenomas, lipomas, cysts, and cancer individually and as a group. Mass characteristics were analyzed across the above groupings for agreement on the single descriptor along with combined analyses where similar descriptors were considered as agreement. These included “round/oval” or “irregular” for mass shape, “circumscribed” or “non-circumscribed” for mass margin, and “anechoic/hypoechoic/complex cystic or solid,” “isoechoic/hyperechoic,” or “heterogenous” for mass echogenicity. Agreement with the pathological diagnosis when available was also performed which was determined by biopsy result when possible. Due to the limited number of biopsies, a mass with a BI-RADS assessment of 2 (100% chance of benignity) was also noted as benign. BI-RADS 3 cases were excluded from the analysis as the pathology was likely benign but not definitively known. Finally, expert analysis of discrepancies in agreement was undertaken by an experienced radiologist.

### Statistical analysis

Throughout, categorical variables are summarized by proportion (95% confidence interval (CI)), and continuous variables are summarized as mean ± standard deviation. For comparison of diagnostic accuracy, pathological diagnosis was used as the gold standard, and sensitivity, specificity, positive predictive value (PPV), and negative predictive value (NPV) were calculated. Agreement on both diagnosis and mass characteristics was quantified using overall agreement (%), as well as Cohen’s kappa. All statistical analyses were performed using MATLAB (R2019b, The Mathworks, Inc., Natick, MA).

## Supporting information

S1 Table Agreement on mass characteristics between S-Detect interpretation of VSI, compared to other interpretations, sub-divided by the masses included in the analysis. (DOCX)
