# Supplementary material for: No sonographer, no radiologist: Assessing accuracy of artificial intelligence on breast ultrasound volume sweep imaging scans
Source: PLOS Digit Health. 2022 Nov 23;1(11):e0000148. doi: 10.1371/journal.pdig.0000148 (PMC9931251; doi:10.1371/journal.pdig.0000148)
Supplement: S1 Table — (DOCX) [file pdig.0000148.s001.docx]

S1 Table. Agreement on mass characteristics between S-Detect interpretation of VSI, compared to other interpretations, sub-divided by the masses included in the analysis. The combined mass shape analysis is between “round/oval” or “irregular.” The combined margin analysis is between “circumscribed” or “non-circumscribed.” The combined echogenicity analysis is between “anechoic/hypoechoic/complex cystic or solid,” “isoechoic/hyperechoic,” or “heterogenous.” SOC = standard of care.

* Cohen’s kappa is undefined due to contingency table having only one entry.

| **Masses Included** | **Metric** | **Comparison Group** | **Overall Agreement (%)** | **Cohen’s kappa** | **P-Value** |
| --- | --- | --- | --- | --- | --- |
| All | Mass Orientation | S-Detect SOC | 75.7% (n=87/115) | 0.31 (0.087-0.53) | p=0.0086 |
|  |  | Expert SOC | 87.8% (n=101/115) | 0.49 (0.24-0.74) | p<0.0001 |
|  |  | Expert VSI | 85.2% (n=98/115) | 0.46 (0.22-0.7) | p<0.0001 |
|  | Mass Shape | S-Detect SOC | 70.4% (n=81/115) | 0.45 (0.29-0.61) | p<0.0001 |
|  |  | Expert SOC | 56.5% (n=65/115) | 0.3 (0.16-0.45) | p=0.0007 |
|  |  | Expert VSI | 54.8% (n=63/115) | 0.26 (0.11-0.41) | p=0.002 |
|  | Mass Shape (combined) | S-Detect SOC | 78.3% (n=90/115) | 0.49 (0.32-0.67) | p<0.0001 |
|  |  | Expert SOC | 70.4% (n=81/115) | 0.37 (0.19-0.55) | p=0.0016 |
|  |  | Expert VSI | 70.2% (n=80/114) | 0.33 (0.14-0.52) | p=0.0028 |
|  | Mass Margins | S-Detect SOC | 54.8% (n=63/115) | 0.18 (0.015-0.34) | p=0.0041 |
|  |  | Expert SOC | 53.9% (n=62/115) | 0.15 (-0.019-0.32) | p=0.016 |
|  |  | Expert VSI | 55.7% (n=64/115) | 0.19 (0.026-0.36) | p=0.0017 |
|  | Mass Margins (combined) | S-Detect SOC | 71.3% (n=82/115) | 0.39 (0.21-0.56) | p<0.0001 |
|  |  | Expert SOC | 67% (n=77/115) | 0.27 (0.082-0.46) | p=0.0036 |
|  |  | Expert VSI | 70.4% (n=81/115) | 0.35 (0.16-0.53) | p=0.00019 |
|  | Mass Echogenicity | S-Detect SOC | 50.9% (n=58/114) | 0.3 (0.16-0.43) | p<0.0001 |
|  |  | Expert SOC | 47% (n=54/115) | 0.22 (0.085-0.35) | p=0.0046 |
|  |  | Expert VSI | 47% (n=54/115) | 0.22 (0.086-0.35) | p=0.0049 |
|  | Mass Echogenicity (combined) | S-Detect SOC | 85.1% (n=97/114) | 0.34 (0.045-0.63) | p=0.00035 |
|  |  | Expert SOC | 96.5% (n=111/115) | 0.73 (0.48-0.99) | p<0.0001 |
|  |  | Expert VSI | 93.9% (n=108/115) | 0.56 (0.24-0.88) | p<0.0001 |
|  | Mass Posterior Acoustic Features | S-Detect SOC | 60% (n=69/115) | 0.41 (0.28-0.54) | p<0.0001 |
|  |  | Expert SOC | 68.7% (n=79/115) | 0.47 (0.33-0.62) | p<0.0001 |
|  |  | Expert VSI | 69.6% (n=80/115) | 0.5 (0.36-0.64) | p<0.0001 |
| All minus fat necrosis, abscess, and sebaceous cyst | Mass Orientation | S-Detect SOC | 77.3% (n=68/88) | 0.37 (0.13-0.61) | p=0.0037 |
|  |  | Expert SOC | 86.4% (n=76/88) | 0.52 (0.26-0.77) | p<0.0001 |
|  |  | Expert VSI | 83% (n=73/88) | 0.46 (0.21-0.71) | p<0.0001 |
|  | Mass Shape | S-Detect SOC | 73.9% (n=65/88) | 0.47 (0.29-0.66) | p<0.0001 |
|  |  | Expert SOC | 59.1% (n=52/88) | 0.3 (0.12-0.47) | p=0.0027 |
|  |  | Expert VSI | 59.1% (n=52/88) | 0.28 (0.1-0.46) | p=0.0024 |
|  | Mass Shape (combined) | S-Detect SOC | 81.8% (n=72/88) | 0.55 (0.35-0.75) | p<0.0001 |
|  |  | Expert SOC | 73.9% (n=65/88) | 0.39 (0.18-0.61) | p=0.0014 |
|  |  | Expert VSI | 75.9% (n=66/87) | 0.41 (0.19-0.63) | p=0.00044 |
|  | Mass Margins | S-Detect SOC | 61.4% (n=54/88) | 0.27 (0.073-0.46) | p=0.00013 |
|  |  | Expert SOC | 63.6% (n=56/88) | 0.26 (0.049-0.46) | p=0.00083 |
|  |  | Expert VSI | 65.9% (n=58/88) | 0.31 (0.11-0.51) | p<0.0001 |
|  | Mass Margins (combined) | S-Detect SOC | 79.5% (n=70/88) | 0.55 (0.36-0.73) | p<0.0001 |
|  |  | Expert SOC | 78.4% (n=69/88) | 0.49 (0.29-0.7) | p<0.0001 |
|  |  | Expert VSI | 83% (n=73/88) | 0.6 (0.42-0.78) | p<0.0001 |
|  | Mass Echogenicity | S-Detect SOC | 55.2% (n=48/87) | 0.35 (0.2-0.5) | p<0.0001 |
|  |  | Expert SOC | 55.7% (n=49/88) | 0.33 (0.17-0.49) | p<0.0001 |
|  |  | Expert VSI | 54.5% (n=48/88) | 0.32 (0.17-0.48) | p<0.0001 |
|  | Mass Echogenicity (combined) | S-Detect SOC | 89.7% (n=78/87) | 0.49 (0.17-0.8) | p<0.0001 |
|  |  | Expert SOC | 100% (n=88/88) | 1 (1-1) | p<0.0001 |
|  |  | Expert VSI | 96.6% (n=85/88) | 0.75 (0.47-1) | p<0.0001 |
|  | Mass Posterior Acoustic Features | S-Detect SOC | 62.5% (n=55/88) | 0.45 (0.3-0.6) | p<0.0001 |
|  |  | Expert SOC | 69.3% (n=61/88) | 0.51 (0.35-0.66) | p<0.0001 |
|  |  | Expert VSI | 70.5% (n=62/88) | 0.54 (0.39-0.69) | p<0.0001 |
| Cancers, cysts, fibroadenomas, and lipomas | Mass Orientation | S-Detect SOC | 80.8% (n=59/73) | 0.45 (0.2-0.71) | P=0.0013 |
|  |  | Expert SOC | 87.7% (n=64/73) | 0.57 (0.3-0.83) | p<0.0001 |
|  |  | Expert VSI | 84.9% (n=62/73) | 0.52 (0.26-0.78) | p<0.0001 |
|  | Mass Shape | S-Detect SOC | 76.7% (n=56/73) | 0.54 (0.34-0.73) | p<0.0001 |
|  |  | Expert SOC | 60.3% (n=44/73) | 0.34 (0.15-0.52) | p=0.0015 |
|  |  | Expert VSI | 60.3% (n=44/73) | 0.32 (0.12-0.51) | p=0.0014 |
|  | Mass Shape (combined) | S-Detect SOC | 86.3% (n=63/73) | 0.65 (0.44-0.85) | p<0.0001 |
|  |  | Expert SOC | 78.1% (n=57/73) | 0.48 (0.25-0.7) | p=0.00039 |
|  |  | Expert VSI | 79.2% (n=57/72) | 0.48 (0.24-0.71) | p=0.00018 |
|  | Mass Margins | S-Detect SOC | 63% (n=46/73) | 0.31 (0.1-0.52) | p<0.0001 |
|  |  | Expert SOC | 64.4% (n=47/73) | 0.29 (0.075-0.51) | p=0.00031 |
|  |  | Expert VSI | 67.1% (n=49/73) | 0.35 (0.13-0.56) | p<0.0001 |
|  | Mass Margins (combined) | S-Detect SOC | 82.2% (n=60/73) | 0.61 (0.42-0.8) | p<0.0001 |
|  |  | Expert SOC | 80.8% (n=59/73) | 0.56 (0.35-0.77) | p<0.0001 |
|  |  | Expert VSI | 84.9% (n=62/73) | 0.65 (0.46-0.84) | p<0.0001 |
|  | Mass Echogenicity | S-Detect SOC | 56.9% (n=41/72) | 0.38 (0.21-0.54) | p<0.0001 |
|  |  | Expert SOC | 57.5% (n=42/73) | 0.36 (0.19-0.53) | p<0.0001 |
|  |  | Expert VSI | 57.5% (n=42/73) | 0.37 (0.2-0.54) | p<0.0001 |
|  | Mass Echogenicity (combined) | S-Detect SOC | 93.1% (n=67/72) | 0.64 (0.34-0.94) | p<0.0001 |
|  |  | Expert SOC | 100% (n=73/73) | 1 (1-1) | p<0.0001 |
|  |  | Expert VSI | 98.6% (n=72/73) | 0.9 (0.71-1.1) | p<0.0001 |
|  | Mass Posterior Acoustic Features | S-Detect SOC | 64.4% (n=47/73) | 0.47 (0.31-0.64) | p<0.0001 |
|  |  | Expert SOC | 75.3% (n=55/73) | 0.59 (0.43-0.76) | p<0.0001 |
|  |  | Expert VSI | 76.7% (n=56/73) | 0.62 (0.47-0.78) | p<0.0001 |
| Fibroadenomas | Mass Orientation | S-Detect SOC | 95.8% (n=23/24) | 0 (-1.9-1.9) | p>0.99 |
|  |  | Expert SOC | 91.7% (n=22/24) | 0 (-1.3-1.3) | p>0.99 |
|  |  | Expert VSI | 95.8% (n=23/24) | 0 (-1.9-1.9) | p>0.99 |
|  | Mass Shape | S-Detect SOC | 87.5% (n=21/24) | -0.043 (-1.1-1.1) | p=0.83 |
|  |  | Expert SOC | 79.2% (n=19/24) | 0.22 (-0.4-0.83) | p=0.35 |
|  |  | Expert VSI | 79.2% (n=19/24) | -0.081 (-0.92-0.76) | p=0.7 |
|  | Mass Shape (combined) | S-Detect SOC | 91.7% (n=22/24) | -0.043 (-1.4-1.3) | p=0.83 |
|  |  | Expert SOC | 79.2% (n=19/24) | -0.071 (-0.91-0.76) | p=0.82 |
|  |  | Expert VSI | 83.3% (n=20/24) | -0.067 (-1-0.89) | p=0.81 |
|  | Mass Margins | S-Detect SOC | 83.3% (n=20/24) | 0.14 (-0.62-0.91) | p=0.42 |
|  |  | Expert SOC | 70.8% (n=17/24) | -0.11 (-0.79-0.58) | p=0.54 |
|  |  | Expert VSI | 79.2% (n=19/24) | -0.071 (-0.91-0.76) | p=0.66 |
|  | Mass Margins (combined) | S-Detect SOC | 87.5% (n=21/24) | 0.33 (-0.37-1) | p=0.13 |
|  |  | Expert SOC | 70.8% (n=17/24) | -0.17 (-0.89-0.56) | p=0.43 |
|  |  | Expert VSI | 79.2% (n=19/24) | -0.11 (-0.98-0.76) | p=0.61 |
|  | Mass Echogenicity | S-Detect SOC | 39.1% (n=9/23) | 0.047 (-0.26-0.36) | p=0.79 |
|  |  | Expert SOC | 45.8% (n=11/24) | -0.058 (-0.45-0.33) | p=0.85 |
|  |  | Expert VSI | 45.8% (n=11/24) | -0.033 (-0.41-0.35) | p=0.91 |
|  | Mass Echogenicity (combined) | S-Detect SOC | 82.6% (n=19/23) | 0 (-0.89-0.89) | p>0.99 |
|  |  | Expert SOC | 100% (n=24/24) | * | - |
|  |  | Expert VSI | 100% (n=24/24) | * | - |
|  | Mass Posterior Acoustic Features | S-Detect SOC | 50% (n=12/24) | 0.16 (-0.18-0.49) | p=0.4 |
|  |  | Expert SOC | 54.2% (n=13/24) | 0.21 (-0.13-0.56) | p=0.53 |
|  |  | Expert VSI | 58.3% (n=14/24) | 0.27 (-0.072-0.62) | p=0.41 |
| Cancers | Mass Orientation | S-Detect SOC | 65% (n=13/20) | 0.3 (-0.12-0.72) | p=0.31 |
|  |  | Expert SOC | 70% (n=14/20) | 0.4 (-0.0017-0.8) | p=0.091 |
|  |  | Expert VSI | 65% (n=13/20) | 0.36 (-0.016-0.74) | p=0.072 |
|  | Mass Shape | S-Detect SOC | 75% (n=15/20) | 0.21 (-0.4-0.81) | p=0.39 |
|  |  | Expert SOC | 70% (n=14/20) | 0.13 (-0.45-0.71) | p=0.51 |
|  |  | Expert VSI | 65% (n=13/20) | 0.067 (-0.49-0.62) | p=0.71 |
|  | Mass Shape (combined) | S-Detect SOC | 75% (n=15/20) | 0.17 (-0.47-0.8) | p=0.57 |
|  |  | Expert SOC | 70% (n=14/20) | 0.077 (-0.54-0.69) | p=0.76 |
|  |  | Expert VSI | 65% (n=13/20) | 0 (-0.6-0.6) | p>0.99 |
|  | Mass Margins | S-Detect SOC | 25% (n=5/20) | -0.02 (-0.28-0.24) | p=0.88 |
|  |  | Expert SOC | 30% (n=6/20) | 0.11 (-0.15-0.36) | p=0.46 |
|  |  | Expert VSI | 30% (n=6/20) | 0.13 (-0.12-0.38) | p=0.41 |
|  | Mass Margins (combined) | S-Detect SOC | 90% (n=18/20) | 0.44 (-0.29-1.2) | p=0.47 |
|  |  | Expert SOC | 90% (n=18/20) | 0.62 (0.11-1.1) | p=0.022 |
|  |  | Expert VSI | 90% (n=18/20) | 0.62 (0.11-1.1) | p=0.022 |
|  | Mass Echogenicity | S-Detect SOC | 60% (n=12/20) | 0.12 (-0.35-0.59) | p=0.68 |
|  |  | Expert SOC | 50% (n=10/20) | 0.091 (-0.31-0.49) | p=0.67 |
|  |  | Expert VSI | 50% (n=10/20) | 0.091 (-0.31-0.49) | p=0.67 |
|  | Mass Echogenicity (combined) | S-Detect SOC | 95% (n=19/20) | 0 (-1.9-1.9) | p>0.99 |
|  |  | Expert SOC | 100% (n=20/20) | * | - |
|  |  | Expert VSI | 100% (n=20/20) | * | - |
|  | Mass Posterior Acoustic Features | S-Detect SOC | 60% (n=12/20) | 0.4 (0.076-0.72) | p=0.022 |
|  |  | Expert SOC | 70% (n=14/20) | 0.5 (0.17-0.84) | p=0.0051 |
|  |  | Expert VSI | 70% (n=14/20) | 0.54 (0.23-0.85) | p=0.00088 |
| Cysts | Mass Orientation | S-Detect SOC | 82.6% (n=19/23) | 0 (-0.89-0.89) | p>0.99 |
|  |  | Expert SOC | 100% (n=23/23) | * | - |
|  |  | Expert VSI | 91.3% (n=21/23) | 0 (-1.3-1.3) | p>0.99 |
|  | Mass Shape | S-Detect SOC | 69.6% (n=16/23) | 0.17 (-0.34-0.68) | p=0.45 |
|  |  | Expert SOC | 26.1% (n=6/23) | -0.048 (-0.3-0.21) | p=0.87 |
|  |  | Expert VSI | 30.4% (n=7/23) | -0.034 (-0.31-0.25) | p=0.91 |
|  | Mass Shape (combined) | S-Detect SOC | 91.3% (n=21/23) | 0 (-1.3-1.3) | p>0.99 |
|  |  | Expert SOC | 78.3% (n=18/23) | 0 (-0.78-0.78) | p>0.99 |
|  |  | Expert VSI | 81.8% (n=18/22) | 0 (-0.89-0.89) | p>0.99 |
|  | Mass Margins | S-Detect SOC | 78.3% (n=18/23) | 0.18 (-0.46-0.82) | p=0.35 |
|  |  | Expert SOC | 87% (n=20/23) | 0 (-1.1-1.1) | p>0.99 |
|  |  | Expert VSI | 87% (n=20/23) | 0.22 (-0.59-1) | p=0.44 |
|  | Mass Margins (combined) | S-Detect SOC | 78.3% (n=18/23) | 0.16 (-0.49-0.81) | p=0.46 |
|  |  | Expert SOC | 87% (n=20/23) | 0 (-1.1-1.1) | p>0.99 |
|  |  | Expert VSI | 91.3% (n=21/23) | 0.47 (-0.24-1.2) | p=0.1 |
|  | Mass Echogenicity | S-Detect SOC | 69.6% (n=16/23) | 0.17 (-0.34-0.68) | p=0.38 |
|  |  | Expert SOC | 82.6% (n=19/23) | 0 (-0.89-0.89) | p>0.99 |
|  |  | Expert VSI | 82.6% (n=19/23) | 0.16 (-0.6-0.91) | p=0.54 |
|  | Mass Echogenicity (combined) | S-Detect SOC | 100% (n=23/23) | * | - |
|  |  | Expert SOC | 100% (n=23/23) | * | - |
|  |  | Expert VSI | 100% (n=23/23) | * | - |
|  | Mass Posterior Acoustic Features | S-Detect SOC | 73.9% (n=17/23) | 0.29 (-0.2-0.78) | p=0.13 |
|  |  | Expert SOC | 95.7% (n=22/23) | 0.83 (0.51-1.2) | p=0.00013 |
|  |  | Expert VSI | 100% (n=23/23) | 1 (1-1) | p<0.0001 |
| Lipomas | Mass Orientation | S-Detect SOC | 66.7% (n=4/6) | -0.2 (-1.6-1.2) | p=0.62 |
|  |  | Expert SOC | 83.3% (n=5/6) | 0 (-1.8-1.8) | p>0.99 |
|  |  | Expert VSI | 83.3% (n=5/6) | 0.45 (-0.52-1.4) | p=0.3 |
|  | Mass Shape | S-Detect SOC | 66.7% (n=4/6) | -0.091 (-1.3-1.1) | p=0.84 |
|  |  | Expert SOC | 83.3% (n=5/6) | 0 (-1.8-1.8) | p>0.99 |
|  |  | Expert VSI | 83.3% (n=5/6) | 0 (-1.8-1.8) | p>0.99 |
|  | Mass Shape (combined) | S-Detect SOC | 83.3% (n=5/6) | 0 (-1.8-1.8) | p>0.99 |
|  |  | Expert SOC | 100% (n=6/6) | * | - |
|  |  | Expert VSI | 100% (n=6/6) | * | - |
|  | Mass Margins | S-Detect SOC | 50% (n=3/6) | -0.29 (-1.3-0.74) | p=0.57 |
|  |  | Expert SOC | 66.7% (n=4/6) | 0 (-1.1-1.1) | p>0.99 |
|  |  | Expert VSI | 66.7% (n=4/6) | 0 (-1.1-1.1) | p>0.99 |
|  | Mass Margins (combined) | S-Detect SOC | 50% (n=3/6) | -0.29 (-1.3-0.74) | p=0.57 |
|  |  | Expert SOC | 66.7% (n=4/6) | 0 (-1.1-1.1) | p>0.99 |
|  |  | Expert VSI | 66.7% (n=4/6) | 0 (-1.1-1.1) | p>0.99 |
|  | Mass Echogenicity | S-Detect SOC | 66.7% (n=4/6) | 0.5 (-0.066-1.1) | p=0.25 |
|  |  | Expert SOC | 33.3% (n=2/6) | 0.14 (-0.34-0.63) | p=0.78 |
|  |  | Expert VSI | 33.3% (n=2/6) | 0 (-0.57-0.57) | p>0.99 |
|  | Mass Echogenicity (combined) | S-Detect SOC | 100% (n=6/6) | 1 (1-1) | p=0.014 |
|  |  | Expert SOC | 100% (n=6/6) | 1 (1-1) | p=0.014 |
|  |  | Expert VSI | 83.3% (n=5/6) | 0 (-1.8-1.8) | p>0.99 |
|  | Mass Posterior Acoustic Features | S-Detect SOC | 100% (n=6/6) | * | - |
|  |  | Expert SOC | 100% (n=6/6) | * | - |
|  |  | Expert VSI | 83.3% (n=5/6) | 0 (-1.8-1.8) | p>0.99 |
